# Lung cancer diagnosis on ovary mass: a case report

**DOI:** 10.1186/1757-2215-6-34

**Published:** 2013-05-10

**Authors:** Nunzia Simona Losito, Cono Scaffa, Monica Cantile, Gerardo Botti, Raffaele Costanzo, Angela Manna, Renato Franco, Stefano Greggi

**Affiliations:** 1 Department of Surgical Pathology, National Cancer Institute “G. Pascale”, Naples, Italy; 2 Department of Gynecologic Oncology, National Cancer Institute “G. Pascale”, Naples, Italy

**Keywords:** Lung adenocarcinoma, Ovarian metastasis, Thyroid transcription factor 1

## Abstract

Metastatic neoplasms to the ovary often cause diagnostic problems, in particular those large ovarian masses mimicking primary tumors. Most of these tumors arise from digestive system or breast, while 37-year-old woman diagnosed as right adnexal complex mass, with a subpleural nodule in the apical part of the left lower lobe, at preoperative chest computed tomography scan. The patient underwent total abdominal hysterectomy with right salpingo-oophorectomy (ovarian mass 220 × 200 mm), total omentectomy, left ovarian biopsy, peritoneal random biopsies, and peritoneal washings for cytology. Pathologic and immunohistochemical examination of ovarian specimen suggested morphology and expression of metastatic lung adenocarcinoma with an intense positivity for Thyroid Transcriptional Factor-1 (TTF-1) and Cytokeratin 7 (CK7) staining. Fine needle biopsy of the lung nodule found epithelioid like malignant cells, confirming the diagnosis of an ovarian metastasis from a primary lung cancer.

This report focused on the clinical and pathologic diagnostic challenge of distinguishing secondary from primary ovarian neoplasms. Issues on useful immunohistochemical stains are also discussed.

## Background

Ovarian complex masses are generally primary carcinoma and less frequently metastasis from extra-gynecological tumors, such as the stomach, colon, breast, pancreas, kidney adenocarcinomas. The occurrence of ovarian metastasis has been reported in a range from 6% to 22%. Most of them are represented by gastrointestinal secondarism [1]. Ovarian metastasis from lung cancer represents only 2-4% of all ovarian metastatic masses [2]. Because of different treatment and prognosis, distinguishing between a primary and metastatic ovarian neoplasm is crucial for correct therapeutic strategy.

In the presence of synchronous tumors, imaging techniques (ultrasound, computed tomography, magnetic resonance), and even conventional morphology are often inadequate for reliable diagnosis. In these circumstances the use of appropriate immunohistochemical markers is able to provide additional evidence to differentiate primary from metastatic neoplasms.

We report a rare case of lung adenocarcinoma with metastasis to the ovary, as the only extra-thoracic localization, and we discuss the clinic-pathologic diagnostic issues in the differential diagnosis, with particular regard to immunoistochemical staining.

## Case presentation

A 37-year-old woman was admitted to Department of Gynaecologic Oncology in March 2009 for a fast-growing pelvic mass and increased serum levels of tumor markers. Her personal oncological history has been characterized by a 1,4 mm melanoma in the left scapular region skin evidenced during the last year, with negative sentinel lymph node and no indications to any postoperative treatment. The patient had undergone appendectomy at the age of 19, and did not have a history of smoking or radiation. Recent Pap-Smear, mammography, and breast ultrasound were negative.

Preoperative work-up included: gynecological examination, transvaginal ultrasound (TVUS), chest-abdomen-pelvis computed tomography (CT) scan, colonoscopy, tumor marker serum levels (CEA, TPA, CA15.3, CA125).

**Figure 1:**
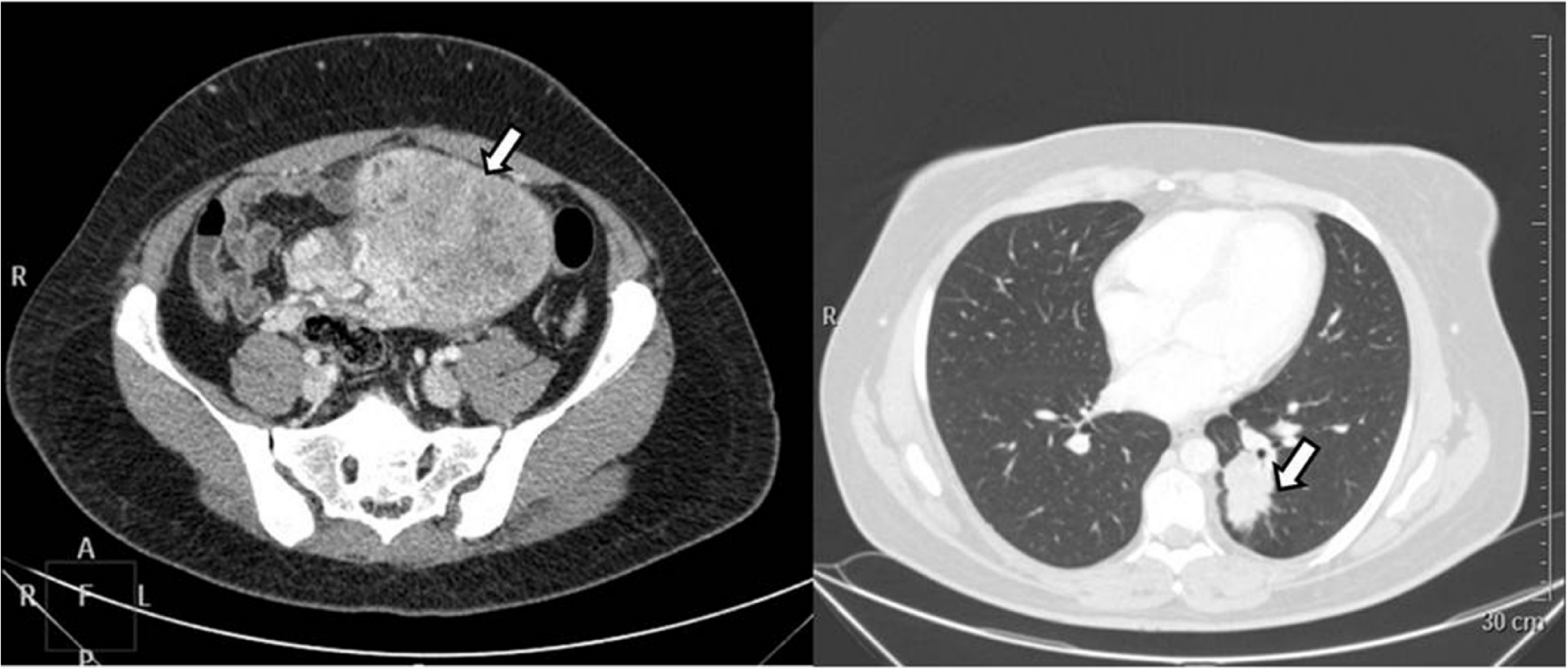
Abdominal-pelvic CT scan (left): parauterine complex mass with necrotic colliquative central area and solid peripheral area (154×108×142mm); thoracic CT scan (right): solid inhomogeneous parenchymal lung tissue in left lower lobe with irregular shape and pleural projections (44×27mm).

CT scan revealed an abdominal-pelvic endoperitoneal complex mass (154×108×142 mm) with a necrotic colliquative central area and a solid peripheral area, displacing ileum and left colon; some aortic lymph node swellings (maximum diameter 14 mm); solid inhomogeneous parenchymal lung tissue in the left lower lobe with irregular shape and pleural projections (44×27 mm); another subpleural nodule in the apical part of the left lower lobe (10 mm) (Figure 1).

Gynecological examination an TVUS confirmed the presence of a gross pelvic mass, likely originating from the adnexa. Colonoscopy was negative for infiltration or endoluminal lesions. Tumor marker serum levels were abnormal: CEA 264.9 ng/ml, TPA 113.0 U/l, CA15.3 499.1 U/ml, CA125 50.5 U/ml.

Therefore, a total body fluorodeoxyglucose - positron emission tomography (FDG-PET) without evidence of accumulation areas further than the abdominal pelvic and lung lesions already described at imaging.

The patient underwent laparotomy with evidence of 20 cm cystic mass arising from right ovary, no lesions of other ovary, nor in the peritoneal cavity; the following procedures were done: total hysterectomy, right salpingo-oophorectomy, total omentectomy, other ovarian peritoneal random biopsies, and peritoneal washings for cytology.

**Figure 2:**
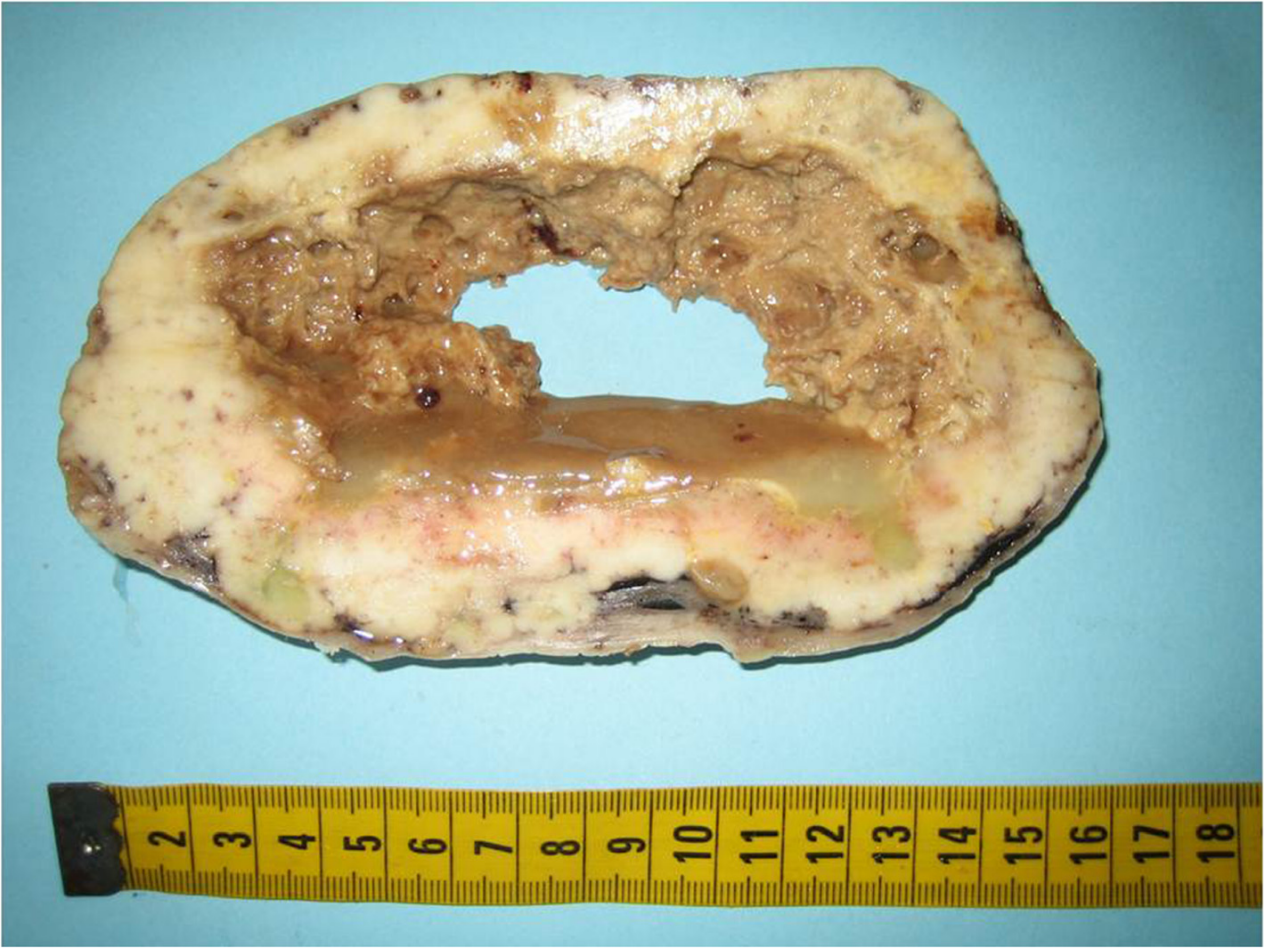
Ovarian metastasis from lung adenocarcinoma: Gross appearance of peripheral solid and whitish rim, with central cystic space mucus containing (cut section).

Intraoperative frozen sections pathology showed a moderately to poorly differentiated, primary or metastatic ovarian adenocarcinoma. Final pathology revealed: 22×20 cm cystic mass from the right ovary with intact capsule and smooth outer surface; the cut surface showed an outlying yellow-pinkish zone with a central yellowish fluid, and the gross appearance at cut section was a peripheral solid and whitish rim, with central cystic space mucus containing (Figure 2); peritoneal cytology, omentum, other ovary, and peritoneal random biopsies negative for malignant cells.

**Figure 3:**
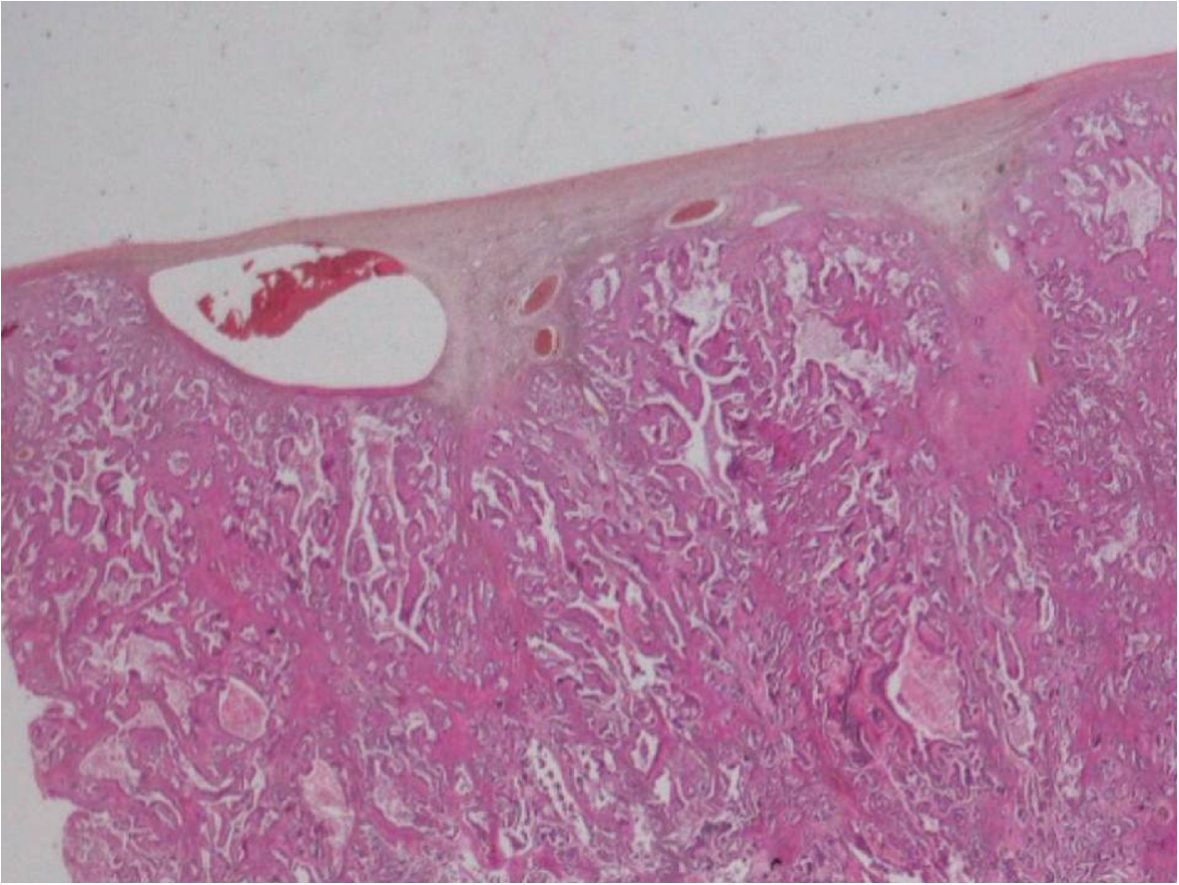
Ovarian metastasis from lung adenocarcinoma: Diffuse ovarian cortex infiltration by moderately differentiated adenocarcinoma (hematoxylin and eosin, original magnification 50×).

**Figure 4:**
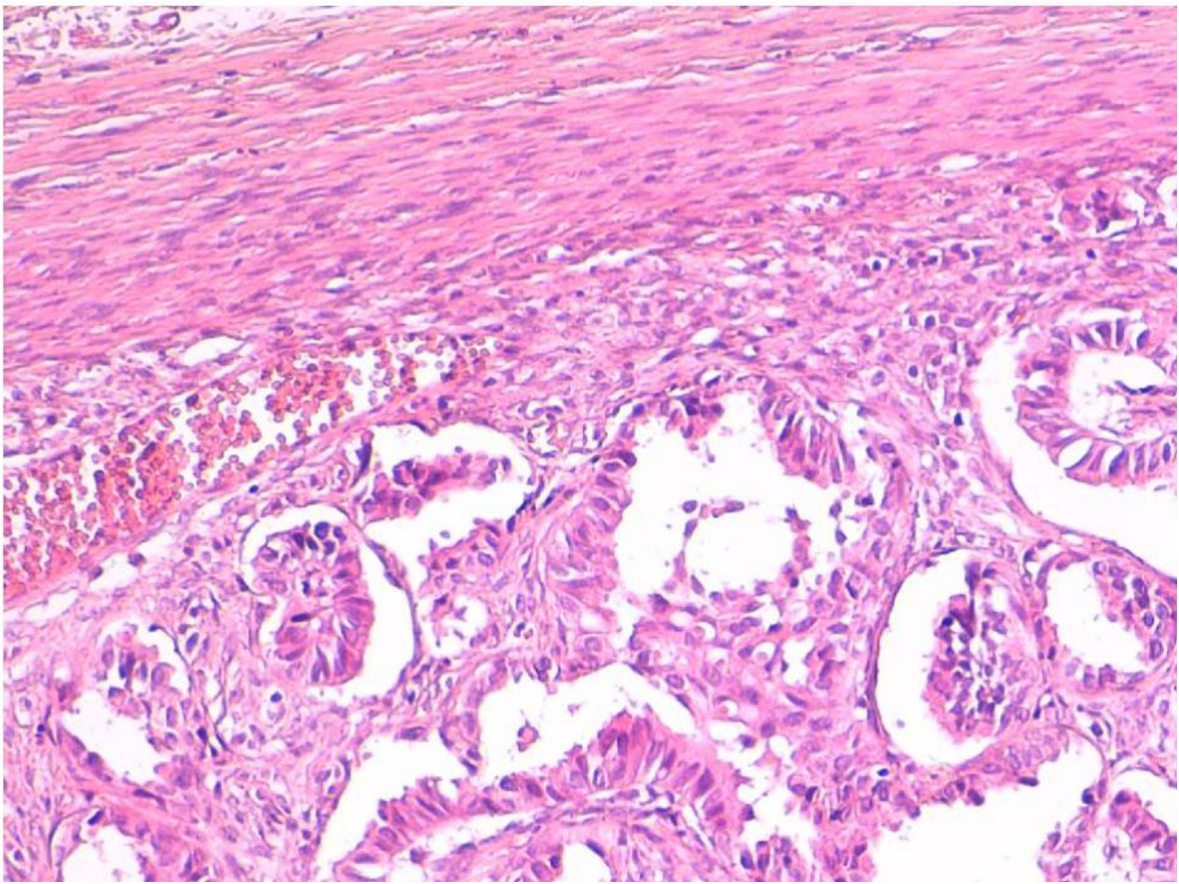
Ovarian metastasis from lung adenocarcinoma: Papillary growth pattern; little neoplastic emboli beneath fibrotic capsule (hematoxylin and eosin, original magnification 250×).

The surgical specimens were fixed in 10% neutral-buffered formalin, routinely processed, and embedded in paraffin. For the light microscopic examination, ovarian tissue sections were cut at a thickness of 4 μm and conventionally stained with hematoxylin and eosin (H&E), periodic acid-Schiff (PAS), and mucicarmin / Alcian blue stains. These histologic permanent sections showed a moderately differentiated papillary adenocarcinoma, with diffuse ovarian cortex infiltration and extensive central necrosis (Figures 3, 4). No resemblance with any conventional primary ovarian adenocarcinoma (serous papillary, endometrioid, clear cell) was observed.

**Figure 5:**
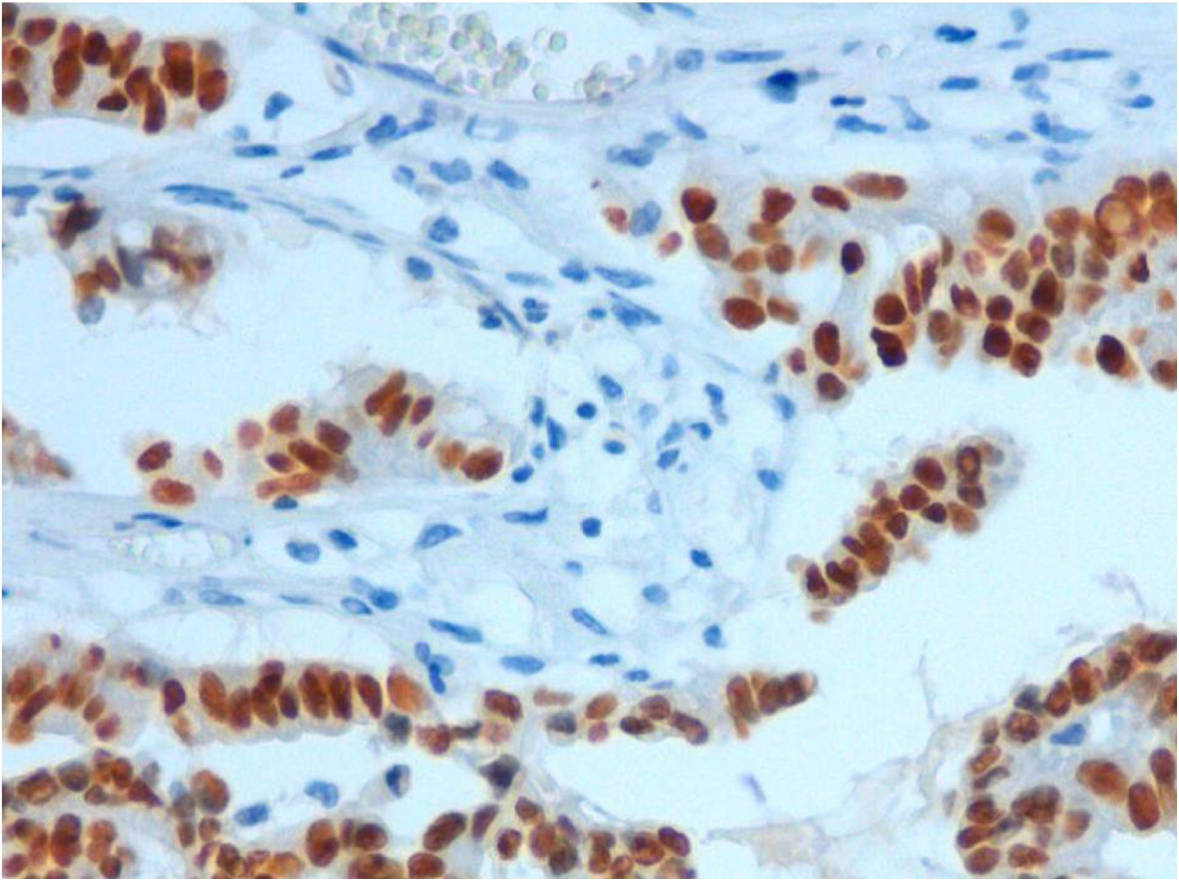
Ovarian metastasis from lung adenocarcinoma: Deep expression of TTF-1 in all neoplastic cells (immunohistochemistry, original magnification 250×).

**Figure 6:**
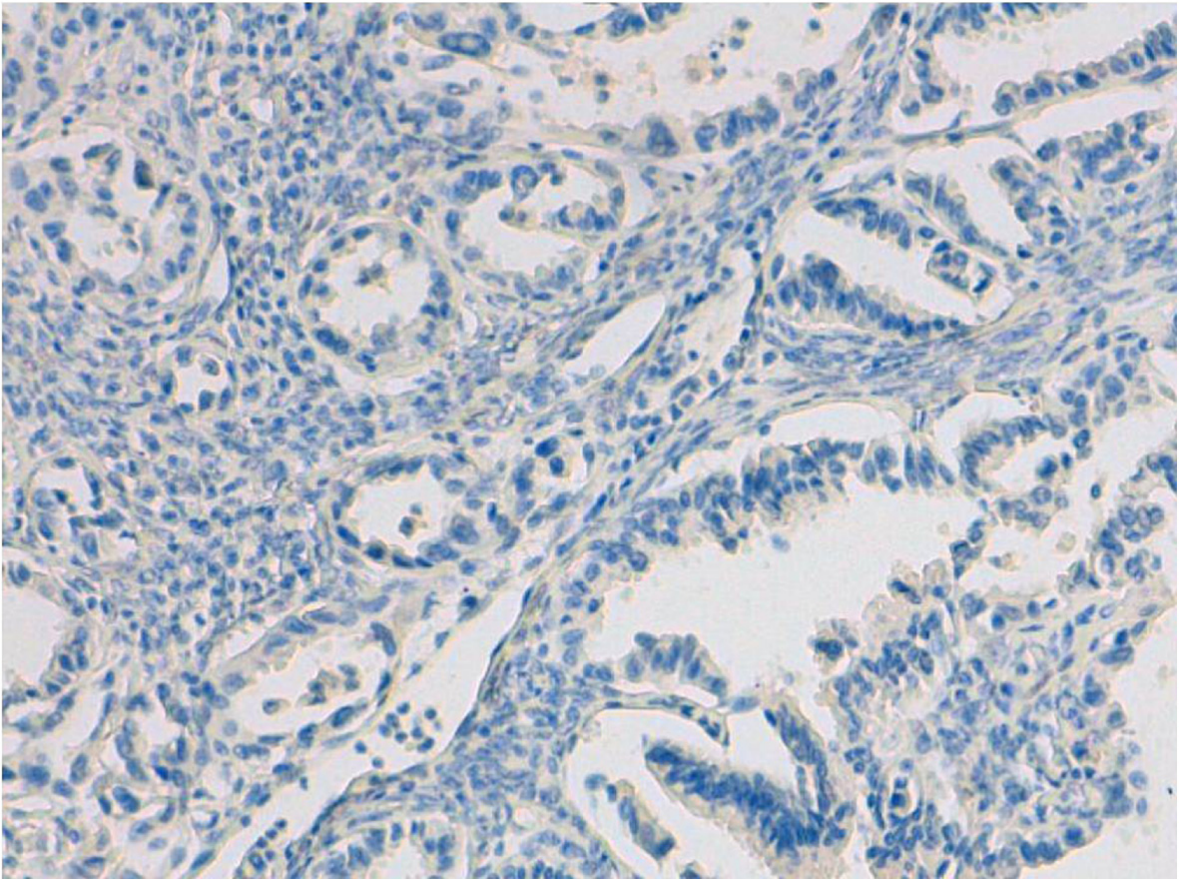
Ovarian metastasis from lung adenocarcinoma: No expression of WT-1 (immunohistochemistry, original magnification 250×).

Immunohistochemistry (IHC) was performed on the 4 μm thick paraffin sections with the avidin-biotincomplex immunoperoxidase technique, using a DAKO Auto Stainer (DAKO, Carpinteria, CA, USA), with EnVision polymer (DAKO, Carpinteria, CA, USA) and specific antibodies (anti-CK7, -TTF-1, -WT-1, -CK20, -CA19-9, -p16). The antigen antibody immunoreaction was visualized using diaminobenzedine (DAB) as a chromogen, and the slides were counterstained with Mayer’s hematoxylin. So, IHC on ovarian specimen was performed: intense positivity for TTF-1 and CK7 staining was found, while WT-1, CK20, CA19-9, and p16 were negative. In particular, a deep expression of TTF-1 and a WT-1 negativity in all neoplastic cells was observed (Figures 5, 6). So, diagnosis of ovarian metastasis from lung adenocarcinoma was suggested.

The postoperative course was uncomplicated, and at one week from surgery the patient was discharged with an adjuvant chemotherapy program. A partial response was achieved after six cycles of carboplatin (AUC 6) and paclitaxel (175 mg/mq) plus bevacizumab (7.5 mg/mq) at day 1 every 21 days, followed by a maintenance therapy with bevacizumab (7.5 mg/mq) every 21 days (stopped for allergic reaction). Afterwards, a stereotaxic radiation therapy was given to the lung lesions (10 Gy in 5 doses), with achievement of a complete remission. In the following ten months there is no evidence of recurrence.

Fine needle guided biopsy (FNB) of the left lung nodule was then performed, showing epithelioid like malignant cells. This study was approved by the ethics committee of National Cancer Institute “G. Pascale”.

## Discussion

The incidence of secondary metastatic tumors in ovary is variable, depending on different factors, such as accuracy of pathological diagnosis, completeness of staging, and possible geographical/genetic patterns. In a study realized on a series of 500 consecutive malignant ovarian neoplasms from Northern America, 17% were metastatic [3], while, in a study from Turkey [4], 22% of 186 ovarian cancers were metastatic. Finally in a very large Chinese study, only 10% of 10288 malignant ovarian neoplasms were metastatic [5].

Ovarian metastases derived from non-gynecologic sites are eleven times more common than those from female genital tract organs, with adenocarcinomas of the gastrointestinal tract being the most frequent [6]. In a recent study on 166 patients with non-gynecologic malignancies and adnexal tumors, 68% of ovarian metastatic tumors were detected [7].

Such tumors may be the initial manifestation of a patient’s cancer, often causing the tumor to be mistaken for a primary ovarian neoplasm, even after microscopic examination. Indeed, a primary extragenital tumor was identified only after surgery for an ovarian mass in 17% of cases in one study [8] and in 38% of patients in another report, with a time interval ranging from 4 days to 7 months [9].

Bilaterality and multinodularity are cardinal features of secondary ovarian carcinomas, irrespective of the site of origin of the primary tumor. In approximately three fourths of cases, both ovaries are grossly involved, although one of the ovaries may be substantially smaller than the other. This is in stark contrast to the very high frequency of unilateral ovarian involvement in primary ovarian tumors of endometrioid, mucinous, and clear cell types, with which metastatic tumors are most often confused. Another feature of secondary ovarian neoplasms is the presence of high-stage disease, affecting multiple peritoneal sites, omentum, and retroperitoneal lymph nodes [1].

Lung carcinoma rarely metastasizes to the ovary, being the primary tumor in only 2-4% of ovarian metastases. Such frequency, however, is increasing due to rising incidence of lung cancer in women [2]. So far, forty cases are been reported of lung cancer metastatic to the ovary in an age range of 26 to 76 years [2,10-16]. Small cell carcinoma and adenocarcinoma were more likely to present with ovarian manifestations than other subtypes (small cell carcinoma: 45% (18 of 40); adenocarcinoma: 32.5% (13 of 40); large cell carcinoma: 12.5% (5 of 40); and 2.5% (1 of 40) for squamous cell carcinoma, atypical carcinoid, bronchiole-alveolar carcinoma, and pulmonary blastoma). A prior lung carcinoma was documented in more than half of cases, the lung and ovarian tumors occurred synchronously in one third, and only in the remaining cases, cancer was first detected in the ovary and then in the lung. Approximately 30% only of ovarian metastases were bilateral. Interesting to note that a coexisting primary ovarian tumor was present in 10% (4/40) of cases.

In the case presented, the ovarian tumor size (22×20 cm), the unilaterality of lesion, the smooth surface with intact capsule, and the absence of intra-abdominal spread were all infrequent features for a metastatic pattern.

Morphologically, we were dealing with a moderately differentiated papillary adenocarcinoma; the papillae and the variably sized glands were embedded in desmoplastic stroma and were lined by a single layer of atypical cuboidal-cylindric cells with apical snouts and huge, hyperchromatic nuclei. Differential diagnosis with primary ovarian carcinoma included the more common histotypes like serous papillary, endometrioid and clear cell adenocarcinoma. Typical broad fibrous papillae and psammoma bodies of serous papillary adenocarcinoma were absent; cylindric and sometimes ciliated cells observed in our case could simulate cells found in endometrioid carcinoma and apical, buldging nuclei could remember, in some way, “hobnail nuclei” typical of clear cell carcinoma, but hyalinized papillae and clear/glycogen rich cytoplasm were both lacking.

In differential diagnosis, IHC is an important adjunct to morphology. In our case, immunohistochemical studies revealed positive staining for TTF-1 and CK7, and negative staining for WT-1, CK20, CA19.9 and p16. So, primary ovarian neoplasm and ovarian metastasis from colonic adenocarcinoma could be excluded.

In particular, TTF-1, member of the NKx2 homeodomain transcription factor family, is expressed in the lung and thyroid and widely used in surgical pathology, also for determining if an adenocarcinoma of unknown primary is of pulmonary origin [17].

This marker has only been studied in a few gynecologic neoplasms and reported in 4% of endocervical adenocarcinomas, 16% of endometrial endometrioid adenocarcinomas, and 23% of endometrial serous carcinomas [18]. Rarely TTF-1 has been found to be expressed in some ovarian tumors but in a recent study, TTF-1 expression significantly improved progression-free (p=0.017) and overall survival (p=0.017), and was identified to be an independent prognostic factor for ovarian cancer in multivariate analysis (p=0.047) [19].

A promising addition on immunohistochemical panel for identifying metastases from lung cancer could be the Napsin A, an aspartic proteinase detected in type 2 pneumocytes and alveolar macrophages, and putative marker for pulmonary adenocarcinomas. Indeed, Napsin A is useful for distinguishing primary lung adenocarcinoma from adenocarcinomas of other organs at primary and metastatic sites [20], and, in particular, the combined use of Napsin A and TTF-1 increased sensitivity and specificity for identifying lung origin in the setting of a metastatic adenocarcinoma [21].

In conclusion, the diagnosis of tumors that secondarily involve the ovary, so as not to mistake them for primary ovarian neoplasms, can cause diagnostic problems, even for experienced gynecologic pathologists and particularly when the primary site is unknown. Ovarian metastases can share both gross and microscopic features with primary ovarian neoplasms, especially for those tumors that produce large, symptomatic ovarian tumors that masquerade clinically and pathologically as primary ovarian tumors of surface epithelial type [Hart, Vang]. In these cases, IHC, with selected immunostains, can be an important adjunctive component in the evaluation of a neoplasm, whether primary or metastatic, with important help to treatment decision making.

Indeed, the current case didn’t show typical clinical and gross features of metastasis, but histopathologic features and immunohistochemical results, notwithstanding occasionally reported TTF-1 expression in ovarian cancer, led us to make the correct diagnosis.

## Consent

Written informed consent was obtained from the patient for publication of this report and any accompanying images.

## Competing interest

The authors declare that they have no competing interests.

## Authors’ contributions

NSL and SG were responsible for the conception and design of the study. CS and RC were responsible for provision of study materials or patient. AM and MC were responsible for immunohistochemical analysis. RF, GB and NSL were responsible for immunohistochemical evalutation. All authors read and approved the final manuscript.
